# Bile Leakage From the Luschka Duct After Laparoscopic Sleeve Gastrectomy and Cholecystectomy: A Case Report

**DOI:** 10.7759/cureus.35684

**Published:** 2023-03-02

**Authors:** Elnur Huseynov, Vusal Aliyev, Gulcan Coban

**Affiliations:** 1 General and Obesity Surgery, Avrupa Safak Hospital, Istanbul, TUR; 2 General Surgery, Bogazici Academy for Clinical Sciences, Istanbul, TUR; 3 General Surgery, Avrupa Safak Hospital, Istanbul, TUR

**Keywords:** biliary duct injury, cholecystectomy, luschka duct, obesity surgery, sleeve gastrectomy

## Abstract

Bile leakage may develop as a result of traumatic or iatrogenic injuries of bile ducts during laparoscopic cholecystectomy (LC). The frequency of Luschka duct injuries during LC is extremely rare. In this case, we present a case of bile leakage due to Luschka duct injury during sleeve gastrectomy (SG) and LC. The leakage was not noticed during the surgery, and on postoperative day 2, bilious drainage was seen from the drain. Magnetic resonance imaging (MRI) was helpful to determine Luschka duct injury. Biliary leakage resolved after endoscopic retrograde cholangiopancreatography (ERCP) with stent placement.

## Introduction

Today, Roux-en-Y gastric bypass (RYGB), sleeve gastrectomy (SG), and adjustable gastric banding are the most popular and commonly performed bariatric surgeries (BS). However, laparoscopic sleeve gastrectomy is considered less technically challenging than laparoscopic RYGB because it does not require a gastrointestinal anastomosis or an intestinal bypass [1]. Laparoscopic cholecystectomy (LC) is one of the most frequently performed operations in surgery departments [2]. The rate of biliary injuries during open and laparoscopic cholecystectomy is 0.1%-0.6% and 0.4%-0.8%, respectively [3]. Biliary leakage may develop as a result of traumatic or iatrogenic injuries of extrahepatic bile ducts, cystic ducts, or sometimes Luschka ducts [4].

The Luschka duct was first described by Hubert von Luschka in 1863, and according to his definition, Luschka ducts are known as subvesical ducts that branch from the right or main hepatic ducts, extend in the submucosa of the posterior wall of the gallbladder, are not connected to the lumen, do not drain any liver parenchyma, and terminate blindly in their distal parts [5]. Postoperative bile leakage may develop due to undetected Luschka duct injury during surgery [6].

Herein, we present a case of biliary leakage due to the Luschka duct after laparoscopic sleeve gastrectomy and cholecystectomy in obese female patients. This case report is in line with the SCARE guidelines [7].

## Case presentation

The 31-year-old female patient decided to undergo an obesity surgery due to suffering for a long time with heavy weight (body mass index {BMI} of 40 kg/m^2^). Her past medical history and preoperative physical examination were unremarkable. During preoperative workup evaluation, multiple gallbladder stones were detected on abdominal ultrasonography (USG). The standard laparoscopic sleeve gastrectomy and cholecystectomy operation was carried out with five ports and Endo GIA (Medtronic Minimally Invasive Therapies, Minneapolis, MN) stapling techniques. The operation went uneventful; no biliary leakage had been seen during the surgery. However, on postoperative day 2, the patient complained of severe right upper abdominal pain, and biliary drainage was observed from the abdominal drain. Her laboratory values were as follows: white blood cell (WBC), 18.7×10^3^/uL; C-reactive protein (CRP), 14.8 mg/dl; aspartate aminotransferase (AST), 38 U/L; alanine transaminase (ALT), 72 U/L; alkaline phosphatase (ALP), 180 U/L; gamma-glutamyl transferase (GGT), 105 U/L; total bilirubin, 1.3 mg/dl; and unconjugated bilirubin, 0.72 mg/dl (Table 1).

**Table 1:** Laboratory value.

	Laboratory value	Normal range
White blood cells (WBC)	18.7×10^3^/uL	4.5-12×10^3^/uL
C-reactive protein (CRP)	14.8 mg/dl	0-5 mg/dl
Aspartate aminotransferase (AST)	38 U/L	0-34 U/L
Alanine transaminase (ALT)	72 U/L	0-55 U/L
Alkaline phosphatase (ALP)	180 U/L	40-150 U/L
Gamma-glutamyl transferase (GGT)	105 U/L	8-36 U/L
Total bilirubin	1.3 mg/dl	0.40-1.35 mg/dl
Conjugated bilirubin	0.72 mg/dl	0-0.5 mg/dl

**Figure 1:**
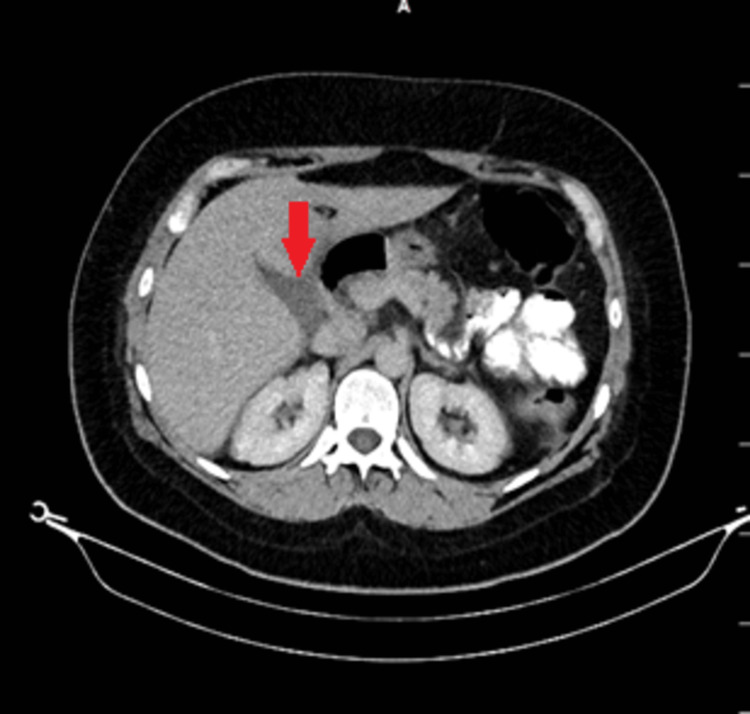
Abdominal CT showed (red arrow) fluid collection on the gallbladder bed. CT: computed tomography

**Figure 2:**
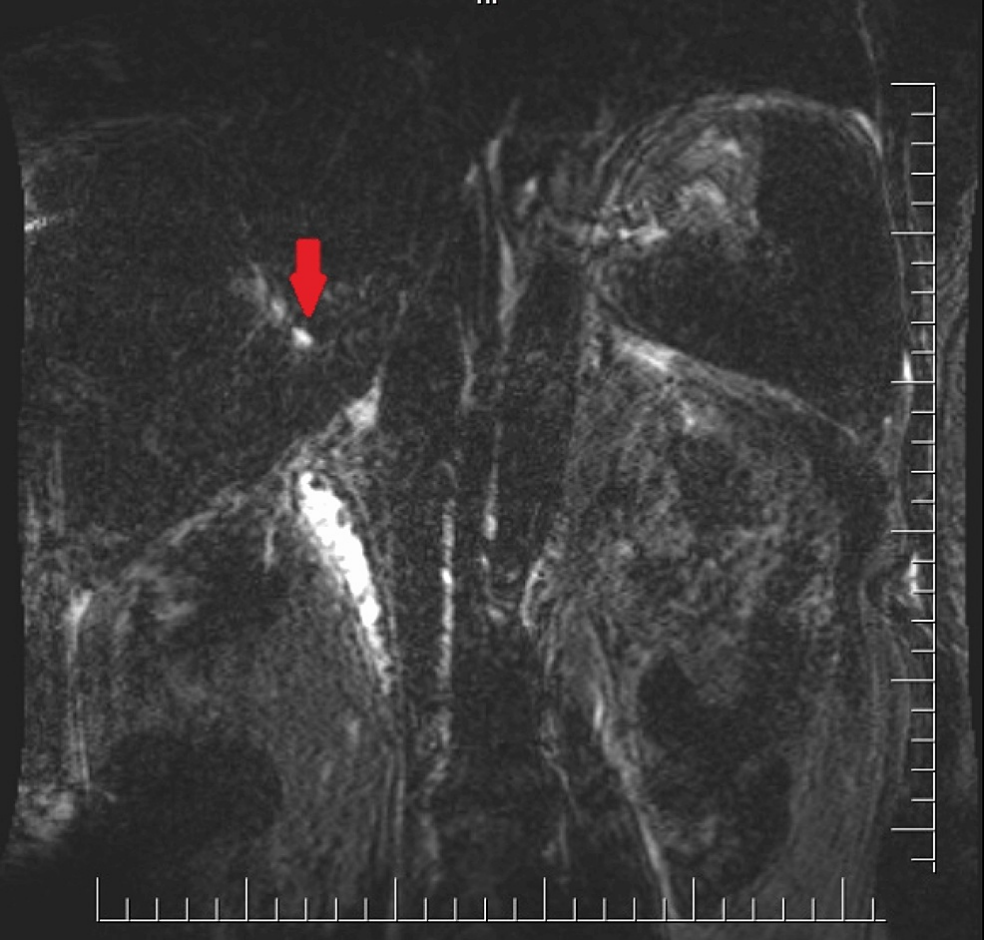
Abdominal MRCP demonstrated (red arrow) the Luschka duct extending into the gallbladder bed. MRCP: magnetic resonance cholangiopancreatography

Oral and intravenous contrasting abdominal computed tomography (CT) was performed and excluded stapling line leakage but showed free fluid on the gallbladder (Figure 1). Magnetic resonance cholangiopancreatography (MRCP) was performed in order to detect the source of biliary leakage. The subvesical bile duct measuring 3 mm in diameter in the liver segment 5 and 8 conjunction opened to the gallbladder was observed (Figure 2). Vital signs were in the normal range. The daily drain bilious output was 600 ml. Endoscopic retrograde cholangiopancreatography (ERCP) was performed due to continuous bilious drainage. Sphincterotomy and biliary stent were placed into choledoch. Subsequently, during the following days, bilious drainage output was decreased. On postoperative day 28, biliary stent was removed, and on day 30, the drain was removed.

The patient feels healthy, and her current BMI is 30.5 kg/m^2^ in postoperative month 8.

## Discussion

The incidence of biliary leakage is rare after LC and associated with abdominal fluid collection, as well as the development of biliary fistula and peritonitis, which may threaten the patient's life [4,8]. Generally, the cause of biliary leakage could be the misplacement of clips, choledoch injury, or leakage from the cystic duct or the Luschka duct [9].

ERCP has been accepted as the gold standard of diagnosis, as well as the management of biliary leakage, and became a primary tool for sphincterotomy and stent placement [10].

Biliary leakage from the Luschka duct after obesity surgery was reported previously [11]. However, it was a mini-gastric bypass surgery patient with cholecystectomy. Due to the altered gastrointestinal tract anatomy, ERCP was impossible in this case. The Luschka duct was clipped via laparoscopy. In our case, we performed laparoscopic sleeve gastrectomy with cholecystectomy, so the gastrointestinal tract anatomy was suitable for the ERCP procedure. To the best of our knowledge, this is the first-ever case report that described postoperative bile leakage from the Luschka duct in a simultaneously performed laparoscopic sleeve gastrectomy and cholecystectomy.

The study with large series concluded that concomitant laparoscopy performed by experienced experts during obesity surgery may prevent future complications associated with gallbladder stones [12,13].

The Luschka duct is an accessory bile duct located in the right lobe of the liver, very close to the gallbladder bed, and empties into the right or common bile duct [14]. Unrecognized Luschka duct injuries may cause delayed peritonitis due to less bile leakage. This type of injury is the most difficult to detect during surgery [15]. If injuries are recognized intraoperatively, duct clipping or liver parenchyma suturing is advised [16]. drain. Magnetic resonance imaging (MRI) is an advantageous noninvasive tool for the detection of bile leakage after LC, as well as helpful for the visualization of the Luschka duct [17-19].

ERCP is a safe and feasible technique for the management of bile leakage after Luschka duct injury [8]. In our case, we saw the closure of the biliary fistula after sphincterotomy and biliary stent placement with ERCP.

## Conclusions

The Luschka duct is an extremely rare biliary duct variation and should be taken into consideration if detected during LC. Otherwise, an injury may lead to bile leakage and furthermore may develop serious clinical conditions. MRCP and ERCP are safe and feasible tools to diagnose and manage bile leakage from the Luschka duct after LC.
